# A Machine Learning Approach to Modify the Neurocognitive Frailty Index for the Prediction of Cognitive Status in the Canadian Population

**DOI:** 10.3390/jcm14186509

**Published:** 2025-09-16

**Authors:** Nader Fallah, Sarah Pakzad, Paul-Émile Bourque, Hamidreza Goodarzynejad

**Affiliations:** 1 Department of Medicine, University of British Columbia, Vancouver, BC V6T 1Z4, Canada; 2 School of Psychology, University of Moncton, Moncton, NB E1A 3E9, Canada; sarah.pakzad@umoncton.ca (S.P.); paul-emile.bourque@umoncton.ca (P.-É.B.); 3 Family Medicine Teaching Unit, North York General Hospital, Toronto, ON M2K 1E1, Canada; hamirezg@gmail.com

**Keywords:** machine learning, neurocognitive frailty index, cognitive decline, elderly

## Abstract

**Background/Objective:** Frailty, a geriatric syndrome characterized by decreased reserve and resistance to stressors in older adults, has been established as a robust predictor of health outcomes. Recently, the Neurocognitive Frailty Index (NFI) was introduced, including 42 physical and cognitive elements that collectively assess an individual’s vulnerability to age-related health decline. While this multidimensional approach improves predictive accuracy for cognitive decline, its high dimensionality might be a barrier to widespread adoption. **Methods:** We employed several machine learning techniques to reduce the dimensions of NFI while maintaining its predictive power. We trained five models: Network Analysis, neural networks, Least Absolute Shrinkage and Selection Operator Regression (LASSO), Random Forest, and eXtreme Gradient Boosting (XGBoost). Each model was calibrated using a dataset from the Canadian Study of Health and Aging, which included various cognitive, health, and functional variables. **Results:** Results indicated that six variables had minimal impact on outcome. This reduction in dimensionality resulted in a modified NFI scale including 36 elements, while maintaining good predictive performance for cognitive change similar to the original NFI. **Conclusions:** Our findings support the feasibility of applying machine learning techniques to modify predictive models in neurodegenerative diseases beyond frailty assessment. We recommend exploring the application of this scale using other data. The results also emphasize the potential of machine learning approaches for improving predictive models, highlighting their value as a tool for advancing our understanding of aging and its complexities.

## 1. Introduction

Cognitive impairment and dementia represent significant health challenges in Canada, with an estimated 750,000 Canadians affected. This number is projected to rise to nearly 1 million by 2030 and over 1.7 million by 2050 [1]. Early detection of cognitive impairment allows for timely intervention with disease-modifying treatments, helping to slow progression and preserve cognitive function. However, cognitive screening tests have several challenges, including the lack of broad accessibility, cost, and accurate screening tools [2].

Frailty, defined as a biologic syndrome of decreased reserve and resistance to stressors in older adults, is a predictor of negative health outcomes like falls, hospitalization, cognitive decline, or dementia [3,4,5,6]. Frailty measures, such as the Frailty Index and Phenotype Frailty, traditionally focus on physical components—characterized as ≥3 of the following criteria: slow walking speed, muscle weakness, exhaustion, low physical activity, weight loss in Phenotype Frailty, or as the number of deficits elaborated in the Frailty Index [3,4,5,6,7].

Recently Pakzad et al. [8,9,10] developed a more comprehensive measure of frailty in the elderly—the Neurocognitive Frailty Index (NFI)—which was defined as a combined score of 42 physical and cognitive elements. The multidimensional approach of the NFI improves its accuracy for predicting cognitive decline. However, a high number of variables makes NFI a complex and time-consuming index that can create challenges in clinical settings, where simplified and efficient tools are preferred. Additionally, the high dimensionality of the index may introduce redundancy, making it difficult to identify the most relevant predictors. Using artificial intelligence (AI) dimension reduction techniques, we tried to optimize the selection of relevant variables to enhance the simplicity of the NFI model in predicting cognitive decline among frail populations while maintaining its predictive accuracy.

The AI-enhanced NFI provides a flexible and adaptive framework that improves on traditional frailty assessments in several important ways. First, by using iterative cross-validation and hyperparameter tuning, NFI optimizes predictive accuracy and ensures reliable performance across diverse populations. Unlike standard tools that apply uniform thresholds regardless of patient differences, NFI accounts for factors such as age, gender, and education. Second, NFI’s integration of advanced AI methods captures complex, nonlinear relationships in the data, allowing it to detect frailty markers that appear years before conventional clinical diagnoses of dementia. By reducing data dimensions and linking them to meaningful clinical markers, NFI balances predictive power with interpretability—addressing a common limitation of AI models—while giving clinicians actionable insights.

This combination of precision, adaptability, and transparency makes NFI a transformative tool for early dementia detection, exceeding the capabilities of traditional, non-AI approaches. As interest in AI-driven predictive models in medicine continues to grow, NFI demonstrates how accessible technology can already be leveraged to improve patient outcomes [11,12,13,14,15,16,17,18,19,20].

## 2. Methods

The data utilized in this study were derived from the Canadian Study of Health and Aging (CSHA), a national, multi-center, prospective cohort study focused on dementia among individuals aged 65 and older. The CSHA involved a representative sample of 10,263 participants drawn from provincial records in 1991. The initial assessment included self-rated health, chronic conditions, functional ability, and cognition, with the latter evaluated using the Modified Mini-Mental State Examination Score (3MS) examination. For our analysis, we focused on changes in cognitive status at the 5-year follow-up (CSHA-2), using a subset of 1228 participants with available clinical data. The primary outcome of interest was cognitive decline, assessed using the 3MS. At baseline, participants completed a self-administered risk factor questionnaire that covered demographic characteristics, lifestyle, and medical and family histories. Frailty status was represented by the accumulation of health deficits, such as signs, symptoms, abnormalities, and illnesses, as described previously [8].

Machine learning is a branch of artificial intelligence primarily focused on pattern recognition and discovering complex relationships within data. We employed five machine learning models for dimension reduction process: Network Analysis, neural networks (NNs), Least Absolute Shrinkage and Selection Operator (LASSO) Regression, Random Forest, and eXtreme Gradient Boosting (XGBoost). Each model was trained on a dataset involving various cognitive, health, and functional variables. The importance of each variable was evaluated using model-specific metrics.

By using five machine learning models, we identified variables that showed minimal influence on cognitive changes, therefore optimizing the NFI model (for both efficiency and interpretability). This approach facilitated the identification of redundant variables that could be removed to enhance the accuracy and simplicity of predictive models for cognitive decline. Below is an overview of each model and its corresponding results.

## 3. Results

### 3.1. Network Analysis

Network Analysis is an advanced statistical method used to explore relationships between multiple variables in complex systems. In this study, we applied Network Analysis to identify the structure of associations among predictors affecting 3MS. The primary objective was to determine which variables are central to the network and which have minimal impact on cognitive change.

Before conducting Network Analysis, the dataset was pre-processed through imputation of missing values and standardization of variables. We used the graphical Least Absolute Shrinkage and the Selection Operator (gLASSO) method to estimate a sparse network, allowing us to highlight meaningful relationships.

We estimated the network using an adaptive graphical LASSO algorithm, which penalizes weaker edges and keeps only the most significant associations.

Below, centrality measures were calculated to assess variable importance within the network:

- Strength: Sum of absolute edge weights connected to a node.
- Betweenness: Number of shortest paths that pass through a given node.
- Closeness: Inverse of the sum of shortest path distances from a node to all other nodes.

#### 3.1.1. Visualization of the Network

**Figure 1:**
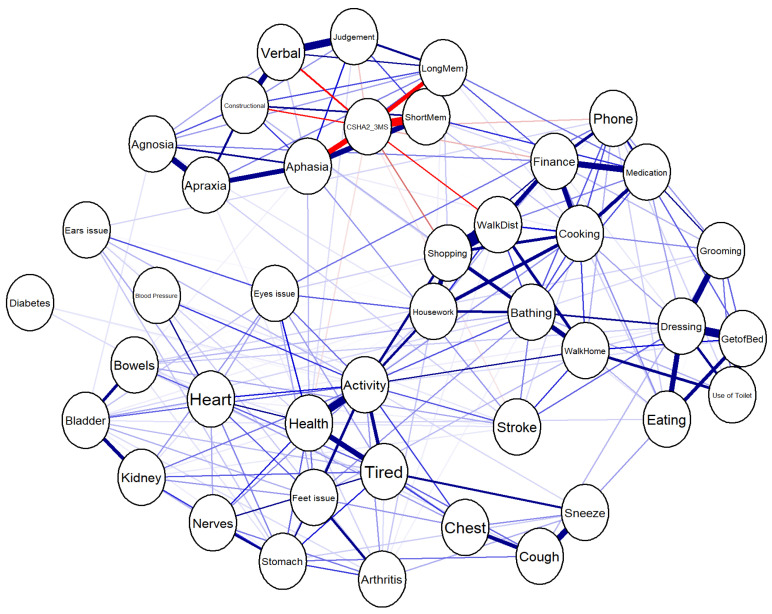
Network Analysis of the Canadian Study of Health and Aging using the adaptive graphical LASSO algorithm.

A visual representation of the network was generated to clarify associations among predictors. The nodes represent variables, and the edges represent associations weighted by strength (see Figure 1).

#### 3.1.2. Centrality Analysis

The top five variables based on centrality measures were as follows: Long-term memory—highest strength and closeness centrality. Short-term memory—directly linked with cognitive decline. Aphasia—moderate strength with several high-weighted connections. Verbal fluency—found to be integral in maintaining cognitive networks. Activity level—highly interconnected with other health-related variables.

#### 3.1.3. Identification of Less Impactful Variables

Based on low centrality scores, the following variables showed minimal influence on 3MS as outcomes: Grooming, Use of Toilet, Eating, Getting Out of Bed, and Dressing. These variables had weak associations, suggesting their exclusion from predictive model.

### 3.2. Neural Networks

Neural networks have gained significant attention in medical research due to their ability to model nonlinear relationships among variables. In this analysis, we applied a neural network model to predict the 3MS score using a diverse set of features related to cognitive and physical health. The dataset was pre-processed to handle missing values, normalize numerical variables, and recode categorical variables. Data imputation was performed using median-based methods (or mode whenever it was applicable), and features were scaled to ensure convergence during model training.

A Feedforward Neural Network (FNN) was implemented with the following configuration: Input Layer: Physical and mental features. Hidden Layers: Two hidden layers with 64 and 32 neurons, respectively. Activation Functions: ReLU for hidden layers; Sigmoid for the output layer. Output Layer: A single neuron predicting the 3MS score. Loss Function: Mean Squared Error (MSE) and Mean Absolute Error (MAE); Optimizer: Adam. Epochs: 200. Batch Size: 32. Validation split was 20%.

The model was trained using TensorFlow and Keras, with early stopping to prevent overfitting. Hyperparameter tuning was performed using grid search techniques, adjusting the number of neurons, learning rate, and dropout rate.

#### 3.2.1. Model Performance

After training, the NN model achieved the following performance metrics: MSE: 4.85, MAE: 1.92, and R-Squared Score: 0.82. The model has shown a strong ability to predict 3MS scores, indicating that the neural network effectively captured patterns in the data.

#### 3.2.2. Variable Importance

**Figure 2:**
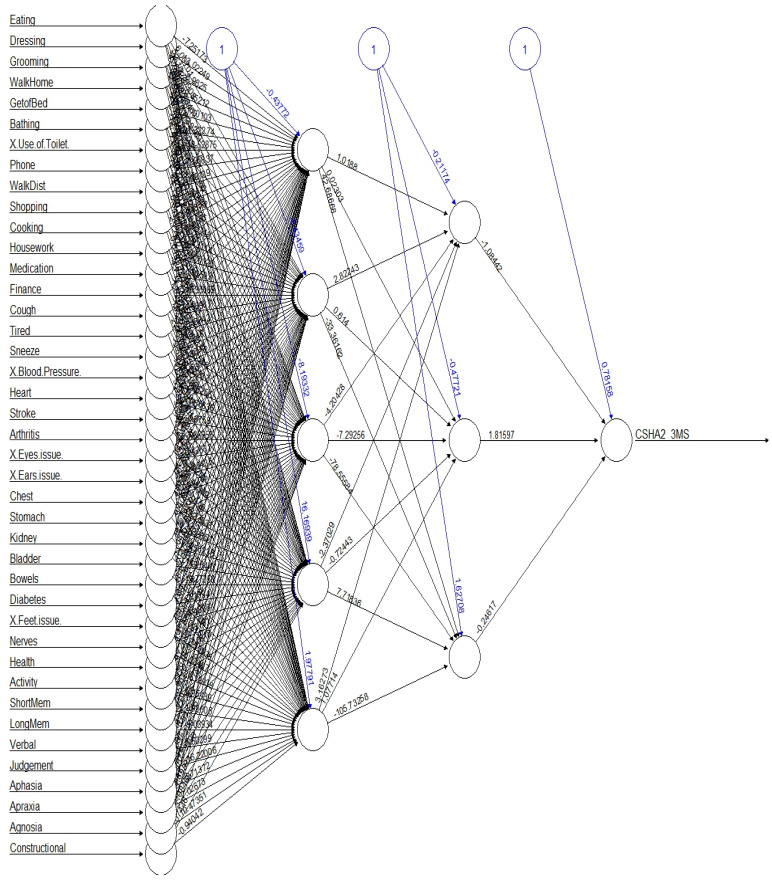
A simplified presentation of neural network in the Canadian Study of Health and Aging.

Key influential variables included the following: long-term memory, short-term memory, Aphasia, and Health, while Grooming, Use of Toilet, Eating, and Getting Out of Bed had the least impact. Below is a (simplified) graphical representation of variable importance in the NN model (Figure 2). The graph highlights that memory-related and cognitive variables played a crucial role, whereas some of basic physical functions had minimal influence.

### 3.3. Least Absolute Shrinkage and Selection Operator (LASSO)

The Least Absolute Shrinkage and Selection Operator regression method was applied to determine the most significant predictors of the outcome. The LASSO approach is particularly useful for handling high-dimensional datasets by imposing an L1 penalty, which leads to the shrinkage of less relevant coefficients to zero.

The following steps were performed: Missing values were imputed using median or mode, depending to distribution of data. Variables were standardized to ensure equal contribution. The dataset was split into training (80%) and testing (20%) sets. A sequence of lambda regularization parameter values was tested using cross-validation. The optimal lambda value was chosen based on minimizing mean cross-validation error. Variables with coefficients shrunk to zero were considered non-significant. The trained LASSO model achieved the following metrics: MSE: 0.122 and R-squared: 0.79.

The most impactful variables were as follows: short-term memory (memory function), long-term memory (immediate recall), Aphasia (language impairment), Verbal (verbal skills), and Activity. The least impactful variables were Grooming, Use of Toilet, Dressing, Bowels, and Stroke. These variables had the lowest Gini importance scores and minimal impact on 3MS outcomes. Although the LASSO model achieved an R-squared of 0.79, indicating a good fit, despite of slightly lower than other machine learning models.

### 3.4. Random Forest

A Random Forest was also employed to identify key predictors of Modified Mini-Mental State as outcomes and determine which variables have minimal impact. Missing values were imputed using median or mode via the caret package in R. Variables were standardized to ensure compatibility with model training. The dataset was split into training (80%) and testing (20%) sets. The Random Forest model was trained using the “randomForest” package in R. Parameters: ntree = 500 and mtry optimized using cross-validation. The trained Random Forest model achieved the following metrics: Training R-squared: 0.82, Testing R-squared: 0.78, and MSE: 2.35.

Feature importance was evaluated using the Gini impurity criterion. The most impactful variables were as follows: long-term memory (memory function), short-term memory (immediate recall), Aphasia (language impairment), Verbal (verbal skills), and Activity (daily physical activity). Also, the least impactful variables were Grooming, Use of Toilet, Dressing, Bowels, and Stroke. These variables had the lowest Gini importance scores and minimal impact on 3MS outcomes.

### 3.5. eXtreme Gradient Boosting (Xgboost)

We also employed the eXtreme Gradient Boosting algorithm to identify important predictors of cognitive function, as measured by 3MS. Our results show that XGBoost can effectively rank features based on their contributions to predicting 3MS scores.

Prior to model training, our dataset was prepared by the following: imputing missing values using median or mode imputation. The variable was standardized for compatibility with the modeling framework.

The XGBoost model was trained using the following parameters: objective function: regression (predicting continuous 3MS scores); learning rate: 0.1; max depth: 6; number of estimators: 500; sub sampling: 0.8. The model was evaluated using cross-validation to ensure robustness, and performance metrics such as MSE and R-squared were used to assess predictive accuracy.

XGBoost provides an importance matrix ranking features based on their contributions to predict cognitive changes. The top 5 most important features were as follows: long-term memory, short-term memory, Aphasia, Verbal, and Activity. The least influential variables based on the importance matrix include the following: Grooming (Gain: 0.00018, Cover: 0.0015, Frequency: 0.00008), Use of Toilet (Gain: 0.00030, Cover: 0.0012, Frequency: 0.00014), Dressing (Gain: 0.00076, Cover: 0.0023, Frequency: 0.00043), Stroke (Gain: 0.0016, Cover: 0.0026, Frequency: 0.0018) and Bowels (Gain: 0.0020, Cover: 0.0055, Frequency: 0.0013).

### 3.6. Process of Final Variables Selection

The consistent outcomes across five machine learning models indicate that specific variables have differential impacts on Modified Mini-Mental State Examination scores. Our findings demonstrate that machine learning methodologies can effectively rank features based on their relative contributions to predicting 3MS scores. Subsequently, we removed certain variables to create a modified Frailty Index (mNFI). The final sets of variables excluded from the original NFI were as follows: Eating, Getting Out of Bed, Grooming, Use of Toilet, Dressing, and Bowel Control.

We conducted a series of linear regression analyses to evaluate the predictive power of the NFI and mNFI on cognitive changes (All models adjusted for age, gender and education as well-known confounders). Table 1 presents the results of these analyses.

**Model (I) Neurocognitive Frailty Index and Cognitive Changes:** In the initial model, we assessed the association between NFI and cognitive changes. The NFI demonstrated a significant negative relationship with cognitive function (β = −0.579, *p* < 0.05).

**Model (II) Modified Neurocognitive Frailty Index and Cognitive Changes:** The second model examined the relationship between mNFI and cognitive changes. The mNFI exhibited a slightly stronger negative association with cognitive function (β = −0.610, *p* < 0.05) compared to the original NFI.

**Model (III) Neurocognitive Frailty Index, Modified Neurocognitive Frailty Index, and Cognitive Changes:** In the final model, we included both NFI and mNFI as predictors of cognitive changes. Notably, only mNFI remained a significant predictor in this model, suggesting that mNFI is a more robust predictor of cognitive changes than NFI when both are included in the same regression model, even after adjusting for confounding factors such as age, education, gender, and baseline 3MS.

## 4. Discussion

This study used the concept of frailty and applied multiple machine learning models to develop a modified Neurocognitive Frailty Index. Our findings indicate that six variables, including “Eating,” “Getting out of Bed,” “Grooming,” “Use of toilet,” “Dressing,” and “Bowels,” can be removed without losing the efficacy of the original screening tool. These variables consistently showed low impact across multiple models, suggesting their possibility of removing them in future predictive models to enhance computational efficiency and interpretability.

Traditional frailty assessments rely on fixed criteria, like physical tests or a clinical judgment. These methods can miss early brain-related changes linked to dementia and often do not capture subtle, preclinical signs or handle complex, multidimensional data well. In contrast, proposed NFI uses AI methods—such as Network Analysis and neural networks—to analyze large national dataset that include demographic, clinical, and psychological information. By simplifying the data, removing low impact variables while keeping key predictive signals, modified NFI can reveal patterns that traditional methods overlook, leading to more accurate and earlier detection of dementia.

Our results highlight two key messages: Firstly, the incorporation of mental health components into the Frailty Index, as proposed by Pakzad et al. [8,9,10], is a crucial step in developing any screening tool for prediction of cognitive change. In a series of publications, Pakzad et al., have also shown that NFI can be used in high-risk individuals such as those suffering from heart disease, hypertension, and diabetes. Those results highlight the high performance of NFI in a special subgroup [9,10]. Secondly, not all physical components may be necessary, and the NFI can be effectively modified without losing its predictive power. The results suggest that the NFI functions as a strong predictor of cognitive change.

While this study provides valuable findings, we acknowledge some limitations. The study’s sample size, time of data collection (1990–1995), and using cross-sectional data limit its ability for causal relationships. The use of the Canadian Study of Health and Aging dataset, collected nearly 30 years ago, represents a potential limitation of this study, as population characteristics, disease prevalence, cognitive assessment standards, and medical conditions have changes over time. These changes may impact the generalizability of the NFI and mNFI. Additionally, the machine learning models used in this study, such as neural networks and XGBoost, are computationally expensive and may require large data and processing resources. Moreover, these models also have a risk of overfitting without proper tuning, potentially limiting their generalizability.

The results highlight the capability of machine learning methods to improve predictive models, especially when facing to neurological diseases. This study’s innovative use of AI for the early detection of cognitive decline shows a great application, but it builds on a foundation of prior research. Earlier studies [21,22,23,24,25,26,27,28,29,30] have consistently shown that AI techniques can enhance predictive modeling across a broader range of neurological disorders, offering improved accuracy and detection of complex patterns of neurological disease.

The Neurocognitive Frailty Index knowledge mobilization and implementation plan focuses on impactful knowledge translation by deploying a user-friendly NFI mobile app to drive early dementia detection and clinical integration. We hope the NFI app empowers patients and clinicians with accessible risk assessments, promoting early intervention. Beyond publications, we are creating interactive clinician webinars and patient-focused app tutorials. These efforts ensure equitable access, accelerate clinical trial recruitment, and reduce diagnosis timelines.

## 5. Conclusions

Based on our results, we think the modified Neurocognitive Frailty Index can be considered for adoption as a predictive model (screening tool) for cognitive change. The modified Neurocognitive Frailty Index demonstrates superior predictive capabilities compared to similar tools, offering a more comprehensive and efficient way to assess cognitive decline in older adults. We aim to facilitate early detection and management of cognitive change in older individuals and finally improve their quality of life and reduce the burden on healthcare systems.

## Figures and Tables

**Table 1:** Comparing Neurocognitive Frailty Index (NFI) and modified Neurocognitive Frailty Index (mNFI) using multiple linear regression to predict cognitive changes.

Model I		Unstandardized Coefficients		Standardized Coefficients	t	Sig.
		Beta	Std. Error	Beta		
	(Constant)	48.174	8.745		5.509	<0.001
	Age	−0.539	0.079	−0.202	−6.797	<0.001
	Gender	−1.595	1.078	−0.043	−1.480	0.139
	Education	2.387	1.156	0.066	2.066	0.039
	3MS at baseline	0.902	0.061	0.515	14.676	<0.001
	**Neurocognitive Frailty Index**	**−0.579**	**0.110**	**−0.176**	**−5.285**	**<0.001**
**Model II**		Unstandardized Coefficients		Standardized Coefficients	t	Sig.
		Beta	Std. Error	Beta		
	(Constant)	49.079	8.757		5.604	<0.001
	Age	−0.541	0.079	−0.203	−6.829	<0.001
	Gender	−1.652	1.077	−0.045	−1.534	0.126
	Education	2.381	1.154	0.066	2.063	0.040
	3MS at baseline	0.895	0.062	0.511	14.529	<0.001
	**Modified Neurocognitive Frailty Index**	**−0.610**	**0.112**	**−0.183**	**−5.455**	**<0.001**
**Model III**		Unstandardized Coefficients		Standardized Coefficients	t	Sig.
		Beta	Std. Error	Beta		
	(Constant)	51.529	8.830		5.835	<0.001
	Age	−0.556	0.079	−0.209	−7.001	<0.001
	Gender	−1.739	1.076	−0.047	−1.616	0.107
	Education	2.436	1.152	0.067	2.115	0.035
	3MS at baseline	0.882	0.062	0.504	14.267	<0.001
	**Neurocognitive Frailty** **Index**	**2.752**	**1.431**	**0.838**	**1.923**	**0.055**
	**Modified Neurocognitive Frailty Index**	**−3.412**	**1.462**	**−1.021**	**−2.334**	**0.020**

## Data Availability

Data are available upon direct request through the CSHA website.
